# Trauma systems in Norway: implementation of national recommendations three years down the line

**DOI:** 10.1186/1757-7241-20-S1-O3

**Published:** 2012-03-22

**Authors:** Thomas Kristiansen, Hans Morten Lossius, Kjetil G Ringdal

**Affiliations:** 1 Department of Research, Norwegian Air Ambulance Foundation, Drøbak, Norway; 2 Faculty of Medicine, Faculty Division Oslo University Hospital, University of Oslo, Oslo, Norway; 3 Department of Surgical Sciences, University of Bergen, Bergen, Norway

## Background

Trauma systems have improved the quality of trauma care during the last four decades. However, the implementation of these principles has been far more difficult to achieve than the mere consensus among health care providers. In 2007 national recommendations for trauma systems in Norway were published. We wanted to assess the implementation of the recommendations three years after their publication.

## Methods

We included all 19 hospitals that received trauma patients in the South-Eastern Norway, excluding Oslo. A telephone interview was conducted between 17. - 21. January 2011. We identified 19 criteria from the 2007 recommendations, these were grouped according topics: trauma teams, material resources, protocols and documentation, and training and competencies for personnel.

## Results

The mean number of implemented criteria was 13 (SD: 2.9; Range: 7-19) and only one hospital fulfilled all criteria. There was no difference in the number of implemented criteria between small and large hospitals 13 vs 14 (P= 0.59) or between hospitals located >/< 125 km from a trauma centre: 13 vs 14 criteria (P= 0.41). The trauma team and material resources criteria were on average implemented in 92% and 97% of the responses, respectively. Criteria for protocols and documentation were implemented in 65% of the responses. For criteria regarding training and competency of personnel only 44% of recommendations were implemented. ATLS courses were required in 53% and 42% of hospitals for surgeons and anaesthesiologists, respectively. Only 32% of hospitals had conducted TNCC courses or equivalent training for nurses.

## Conclusion

Three years after the publication of nationally accepted recommendations for trauma management, there are still great deficiencies in the resources of trauma receiving hospitals. There are no accreditation systems of hospitals that make implementation obligatory. Research on effective strategies for implementation deserves further attention.
